# Emerging Understanding of Emotional Intelligence of Teenagers

**DOI:** 10.5005/jp-journals-10005-1452

**Published:** 2017-02-27

**Authors:** Punya Sekhri, Meera Sandhu, Vinod Sachdev

**Affiliations:** 1 Postgraduate Student, Department of Pedodontics and Preventive Dentistry, I.T.S Centre for Dental Studies and Research, Muradnagar, Uttar Pradesh, India; 2 Professor, Department of Pedodontics and Preventive Dentistry, I.T.S Centre for Dental Studies and Research, Muradnagar, Uttar Pradesh, India; 3 Professor and Head, Department of Pedodontics and Preventive Dentistry, I.T.S Centre for Dental Studies and Research, Muradnagar, Uttar Pradesh, India

**Keywords:** Bar-on emotional intelligence inventory, Emotion, Emotional intelligence, Teenagers.

## Abstract

**Aim:**

Emotional intelligence (EI) is the ability to use emotions effectively and productively. It is becoming increasingly clear that these skills are one of the primary foundations for better performance of students in classrooms and in the society as well and EI provides the basis for competencies important "in almost every job." So we accessed the EI of teenagers as a guide of their academic score.

**Study design:**

We analyzed the correlation of academic score to the EI of teenagers in regular schools and part-time unconventional coaching institute using the Bar-On Emotional Quotient questionnaire.

**Results and conclusion:**

The results of our study showed that empathy and self-actualization were highly developed in students of regular conventional school than those attending part-time unconventional coaching institute. The academic score had a significantly positive correlation with empathy, whereas a significantly negative correlation with interpersonal relations. Empathy, interpersonal relation, and impulsive control were significantly higher in females than males. Therefore by inculcating and working toward development of EI in the young generation, we can hope to achieve a more positive environment.

**How to cite this article:**

Sekhri P, Sandhu M, Sachdev V. Emerging Understanding of Emotional Intelligence of Teenagers. Int J Clin Pediatr Dent 2017;10(3):289-292.

## INTRODUCTION

Emotion is an important ability that allows us to tune into how someone is feeling or what they might be thinking. It allows us to understand the intentions of others, predict their behavior, and experience an emotion triggered by their emotion. In short, emotion enables us to interact effectively in the social world. It is the "glue" of social world that draws us to help others and stops us from hurting others.^1^

There are a number of general cultural influences that serve as a context for our thinking about the relation between emotion and cognition. The concept of thought and emotion in Western culture goes back to more than 2000 years, when the Greek’s idea was that reason was superior to emotion (Payne, Solomon). The European Sentimentalist movement’s idea stated that there existed innate, pure, emotional knowledge (Reddy), while the Romantic movement emphasize on emotional expression in the arts (Solomon).^2,3^ During the political turmoil of the 1960s, the public discussion elicited proper balance between feelings and thought.^4,5^

In modern psychology (e.g., Leeper, Young) and philosophy (Desousa), the debate is on relative importance and rationality of emotion and cognition. A new area of recent interest has been the impact of social and emotional competency on academic achievement.

Emotional intelligence is the capacity to reason about emotions as they enhance thinking. It includes the abilities to accurately perceive it, access, generate and to assist thought, to understand emotion and emotional knowledge and to effectively regulate them so as to promote emotional and intellectual growth (Mayer and Salovey).^6^ From theoretical perspective, EI refers specifically to the cooperative combination of intelligence and emotion. Emotional intelligence can be viewed as a member of a class of intelligences, including the social, practical, and personal intelligences.^4^

Early discussions on the relationship between EI and achievement in educational contexts claim an associa-tion.^7^ We hypothesized that students whose EI was more would be more competent academically and socially.^6^

It has been claimed by others that a "considerable body of research" suggests that EI provides the basis for competencies important "in almost every job" (Cherniss).^5^ Therefore this study was conducted to analyze the relationship between EI and academic success of teenagers.

## MATERIALS AND METHODS

In the present study, the sample consisted of 200 students from regular schools and part-time unconventional coaching institutes in Delhi. The students ranged from 14 to 17 years of age with the mean age of 16.21 years and mean academic score of 9.5. This age group was selected as it is the time when an individual’s personality shaping takes place and also because not many studies have been conducted using this age group. To control the effect of cultural differences in emotional expression, participants were excluded if their first language was not English.

A brief introduction on the purpose and intent of the study was given. Consent from the school authorities and parents was taken and the questionnaires were distributed. The students completed the Bar-On Emotional Quotient questionnaire (Bar-On and Parker).^8^ The questionnaire was prepared under the expert guidance of a psychologist.

The understanding of EI of children and adolescents was done by investigating the psychometric properties (i.e., validity) of the Emotional Quotient Inventory: Youth Version (EQ-i: YV). Validation of this quotient involved considering its relationship to cognitive intelligence and self-report of personality. It has a set of scales and subscales which aid in getting a better view of one’s personality.

Many theorists have operationalized their theories of EI with evaluative measures for use. But only the Bar-On EQ-i has evidenced adequate reliability and some degree of validity as stated by Dawda and Hart^9^; therefore, it was used in our study.

## RESULTS

A cross-sectional study was conducted in which 250 questionnaires were distributed to teenagers in regular schools and part-time unconventional coaching institute. Out of 250 forms only 200 which were completely filled were included in the study. The data collected was analyzed using statistical software packages, Statistical Package for the Social Sciences software for Windows (version 16.0); t-test for two independent groups was used to test the significance of the difference of means of parameters between the two groups. The correlation between variables was calculated using Pearson’s correlation. The level of significance was 0.05 (two-tailed) with 95% confidence interval.

The results of our study showed that empathy and self-actualization were highly developed in students of regular conventional school than those attending part-time unconventional coaching institute (Table 1).

**Graph 1:**
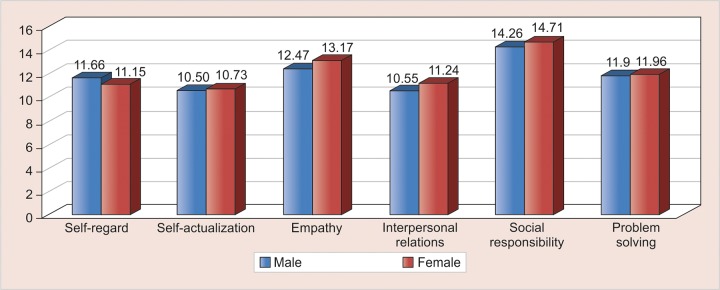
Comparison of different variables of EI between males and females

**Graph 2:**
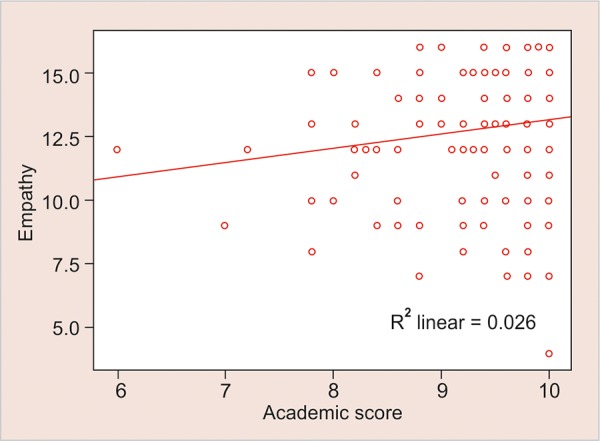
Positive correlation between empathy and academic score

Present study also revealed that academic score had a significantly positive correlation with empathy, whereas a significantly negative correlation with interpersonal relations with the p-values being +0.022 and -0.038 respectively (Graphs 1 and 2).

Empathy, interpersonal relation, and impulsive control were significantly higher in females than in males and other variables showed nonsignificant mean difference, the p-value being 0.044, 0.007, 0.031 for empathy, interpersonal relations, and impulse control respectively (Table 2).

**Table 1:** **Table 1:** Comparison of mean score of EI variables in teenagers attending regular school and coaching institute

*EI variables*		*Institute*		*n*		*Mean ± SD*		*t-value*		*p-value*	
Self-actualization		Regular school		157		10.80 ± 1.72		2.053		0.041	
		Part-time unconventional coaching institute		42		10.19 ± 1.60					
Empathy		Regular school		157		13.04 ± 2.35		1.999		0.047	
		Part-time unconventional coaching institute		42		12.19		2.75					

SD: Standard deviation

**Graph 3:**
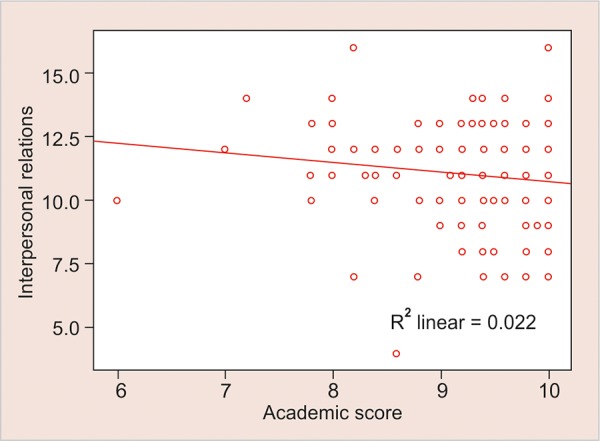
Negative correlation between interpersonal relations and academic score

**Table 2:** **Table 2:** Comparison of mean score of EI variables between male and female

*EI variables*		*Gender*		*n*		*Mean ± SD*		*t-value*		*p-value*	
Empathy		Male		88		12.47 ± 2.68		2.024		0.044	
		Female		111		13.17 ± 2.24					
Interpersonal		Male		88		10.55 ± 2.00		2.725		0.007	
relations		Female		111		11.24 ± 1.61					
Impulse		Male		88		11.00 ± 2.88		2.172		0.031	
control		Female		111		11.85 ± 2.61					

SD: Standard deviation

## DISCUSSION

The term EI is relatively new, which targets at supplementing the conventional view of intelligence by laying emphasis on emotional, personal, and social contributions to intelligent behavior as given by Gardner; Mayer^10^ and Salovey;^6^ Wechsler.^11^ Emotional intelligence is emerging as a crucial factor in order to sustain high achievement, retain positive behavior as well as improving life success. It comprises a set of capabilities that enable an individual to manage oneself and others around. The EQ-i has a set of scales and subscales which are: (1) Intrapersonal skills which include: (a) Emotional self-awareness, (b) assertive-ness, (c) self-regard, (d) self-actualization, and (e) independence; (2) Interpersonal skills which include: (a) Empathy, (b) interpersonal relationship, (c) social responsibility; (3) Adaptation which includes: (a) Problem-solving, (b) reality testing, (c) flexibility; (4) Stress management which includes: (a) Stress tolerance, (b) impulse control; and (5) General mood which includes: (a) Happiness and (b) optimism.^7^

Educational organizations are picking up this concept of EI, in hope of achieving a systemic solution to improve outcomes - academically and socially as well. Finn and Rock in 1997^12^ stated that high school students who exhibit behaviors consistent with social and emotional competency are more apt to be successful in school.^1^

In this study an emerging understanding of EI of teenagers was assessed and EI was compared with the academic scores of teenagers attending regular schools and part-time unconventional coaching institute.

Results of the present study show that empathy, interpersonal relations, and impulse control were significantly higher in females than in males (p-value <0.05, Table 2). This was in accordance with the study conducted by Grewal and Salovey in 2005,^13^ augmenting the stereotype that female gender is more emotional. By nature, emotional dimension of human race has been linked to a greater extent with the female gender as they tend to experience positive and negative emotions more intensely than the male gender.

Empathy is an important ability, it allows one to know how the other person is feeling or what they might be thinking. This emotional variable helps us in understanding the intentions of others, predict their behavior, and to experience an emotion triggered by their emotion. Cognitive and affective approaches are the essentials which define empathy and cannot be easily separated. The observer’s emotional response to the affective state of others and understanding other’s feelings as per the cognitive theories, both are inter-related.^14^ The present study shows that empathy has a significant positive correlation with the academic scores of teenagers but on the contrary interpersonal relations have a significantly negative correlation with the academic score (Graphs 2 and 3).

Interpersonal relationships develop later in life. It is during middle childhood and adolescence that students spend more time with friends. It is a stage where adolescents show an increase in intimacy between friends of opposite gender and focus on sharing common activities. Gradually as an individual moves into young adulthood, friendship becomes more connected to the work environment. The focus shifts from more of social orientation to developmental tasks and there is a better understanding in handling relationship with those around.^5^

The results of this study showed a significantly negative correlation of interpersonal relations with academic score in contrast to earlier studies, which could be because this study included mainly teenagers whereas previous studies included adults also, as in the study conducted by Cadman and Brewer.^15^ The discrepancy in findings for interpersonal abilities could also be as a result of the changing role of the peer group as students move from late adolescence to young adulthood as stated by Hartup and Stevens.^16^ Also, developmental changes in emotional understanding, such as interpersonal skills generally increase with age. Therefore, it would be expected that when students are in university their interpersonal skills will be more developed and would be more predictive of their academic score.^7^

Empathy and self-actualization were seen to be highly developed in students of regular conventional schools than those of part-time unconventional coaching institute (p-value < 0.05, Table 1). The inference that can be drawn from this finding is that regular schools emphasize on overall development of the student. They focus on all aspect of personality development unlike the part-time coaching institute, whose main aim is to train the students to deliver the desired results. As stated by Finn and Rock,^12^ high school students who exhibited behavior consistent with social and emotional competency were more apt to be successful in school.

Therefore, in this time of budget cuts, intense societal pressure on youth, the need of the hour is to look for innovative approaches to address the academic, social, psychological, and physical health needs of developing students. Emotional intelligence prevention and intervention programming may be the key investment which would secure a positive future for the generations to come.^17^

The limitation of this study would be that the study population belonged to the same socioeconomic status. The variability attributed to differences in economic status was not taken into consideration. Also, the cultural and regional disparity was not taken into account. Although not many studies have been conducted to access the emotional quotient of teenagers owing to the fact that their experiences in life and time would alter their perspective, the present study revealed conclusive results with significant differences.

## CONCLUSION

Therefore, early understanding and implementation of EI in children will enhance the overall development of their personality and its use to manage their behavior, and perceptions will create a more positive environment for the human race.
